# Molecular basis and evolutionary drivers of endosperm-based hybridization barriers

**DOI:** 10.1093/plphys/kiae050

**Published:** 2024-01-31

**Authors:** Heinrich Bente, Claudia Köhler

**Affiliations:** Department of Plant Reproductive Biology and Epigenetics, Max Planck Institute of Molecular Plant Physiology, Potsdam 14476, Germany; Department of Plant Reproductive Biology and Epigenetics, Max Planck Institute of Molecular Plant Physiology, Potsdam 14476, Germany; Department of Plant Biology, Swedish University of Agricultural Sciences and Linnean Center for Plant Biology, Uppsala 75007, Sweden

## Abstract

The endosperm, a transient seed tissue, plays a pivotal role in supporting embryo growth and germination. This unique feature sets flowering plants apart from gymnosperms, marking an evolutionary innovation in the world of seed-bearing plants. Nevertheless, the importance of the endosperm extends beyond its role in providing nutrients to the developing embryo by acting as a versatile protector, preventing hybridization events between distinct species and between individuals with different ploidy. This phenomenon centers on growth and differentiation of the endosperm and the speed at which both processes unfold. Emerging studies underscore the important role played by type I MADS-box transcription factors, including the paternally expressed gene *PHERES1*. These factors, along with downstream signaling pathways involving auxin and abscisic acid, are instrumental in regulating endosperm development and, consequently, the establishment of hybridization barriers. Moreover, mutations in various epigenetic regulators mitigate these barriers, unveiling a complex interplay of pathways involved in their formation. In this review, we discuss the molecular underpinnings of endosperm-based hybridization barriers and their evolutionary drivers.

## Teaser

Recent discoveries reveal the role and genetic basis of hybridization barriers established in the endosperm.

## Introduction

Angiosperms comprise approximately 300,000 extant species and represent the most species-rich plant clade, accounting for approximately 90% of all land plant species (Christenhusz and Byng 2016; Katz 2018). Their rapid rise to ecological dominance in the mid-Cretaceous remained the “abominable mystery” to Charles Darwin, as evident from his 1879 letter to Joseph Hooker (Darwin and Seward 1903). Understanding this enigmatic rise to prominence has remained a central objective in evolutionary biology, necessitating insights into the evolution of reproductive barriers that limit gene flow between populations. Much previous research has focused on comprehending prefertilization reproductive barriers, which prevent successful zygote formation through mechanisms like pollinator adaptations or pollen–pistil incompatibilities (Widmer et al. 2009; Hernández-Hernández et al. 2021). Recent advances in the field of evolutionary biology have recognized postzygotic mechanisms as substantial drivers of plant speciation (Coughlan 2023). These mechanisms operate at various time points after fertilization, leading to impaired viability of hybrid seeds or seedlings or sterility of the resulting hybrids (He et al. 2023). In this review, we focus on the evolution of postzygotic barriers arising from the endosperm, particularly in the context of interspecies and interploidy hybridizations. We discuss the molecular mechanisms underlying these barriers and explore their impact on plant speciation.

## Endosperm development

Seed development in flowering plants starts with double fertilization, whereby 1 of the 2 pollen-delivered sperm cells fertilizes the egg cell, initiating the formation of the embryo, and the other sperm cell fertilizes the central cell, giving rise to the endosperm (Dresselhaus et al. 2016). The endosperm is a unique feature of angiosperms, playing a crucial role as a nourishing tissue to support the growth and germination of the embryo (Doll and Ingram 2022). In most angiosperms, the endosperm is a triploid tissue with 2 maternal and 1 paternal genome copies. This triploid constitution arises since the central cell is a homodiploid cell (Dresselhaus et al. 2016). This increased maternal copy number may have evolved to regulate resource allocation to the embryo, possibly reducing the influence of paternally provided growth-promoting alleles and ensuring equal provisioning of seeds (Haig and Westoby 1989). The ancestral state of the endosperm, observed in sister lineages to all angiosperms such as Nymphaeales and Austrobaileyales, is diploid. Resource allocation in these species is typically mediated by the perisperm, a nutritive tissue derived from the maternal sporophyte (Floyd and Friedman 2000; Williams and Friedman 2002).

**Advances**

- Genetic data substantiate a model wherein AGLs function upstream of a regulatory cascade governing endosperm cellularization, making understanding of *AGL* regulation and their downstream targets of crucial relevance.
- Maternal Pol IV–dependent siRNAs play a pivotal role in suppressing *AGL* expression, thus fostering endosperm cellularization. Conversely, paternal Pol IV–dependent siRNAs exert a negative influence on endosperm cellularization, illustrating the antagonistic functions of maternal and paternal siRNAs in this process.
- The inhibitory effect of auxin on endosperm cellularization underscores the importance of unraveling auxin signaling within the endosperm.
- Endosperm cellularization serves to protect the embryo from desiccation, a process typically occurring during maturation. The precise role of ABA in this process remains in important open question.

Endosperm development leads to the formation of a fully cellularized tissue surrounding the embryo. However, the process of endosperm cellularization is not uniform across all species. In most angiosperms, the endosperm does not become cellular from the start; instead, nuclear divisions occur initially without cellularization, resulting in the formation of a coenocyte. Subsequently, cellularization takes place after a defined number of mitotic cycles, followed by the differentiation of distinct cell types (Boisnard-Lorig et al. 2001; Sorensen et al. 2002; Berger 2003). This developmental transition is critical for embryo survival; impaired endosperm cellularization in Arabidopsis (*Arabidopsis thaliana*) mutants is generally connected with embryo arrest (Grossniklaus et al. 1998; Kiyosue et al. 1999; Pignocchi et al. 2009; Hehenberger et al. 2012). In contrast to the nuclear type of endosperm development, certain genera like *Solanum*, *Mimulus*, and *Nicotiana* exhibit the cellular endosperm type, where nuclear division and cellularization are coupled events (Lopes and Larkins 1993; Floyd and Friedman 2001). Additionally, an intermediate type of endosperm development exists, known as the helobial endosperm. After the first division of the fertilized central cell, 1 cell follows the nuclear type of development while the other follows the cellular type, resulting in a unique helobial endosperm. Helobial endosperm development is relatively rare but can be found in species of the Cabombaceae, Sabiaceae, and Saxifragaceae families (Geeta 2003).

The endosperm plays a crucial role as a nutrient reservoir for the embryo, and its reserves can be remobilized during embryo development and germination. While in monocots the endosperm is preserved in the mature seed, serving to support embryo germination, in most eudicots, the endosperm is consumed by the developing embryo as it grows (Li and Berger 2012).

## The endosperm establishes reproductive isolation

**Figure 1.**
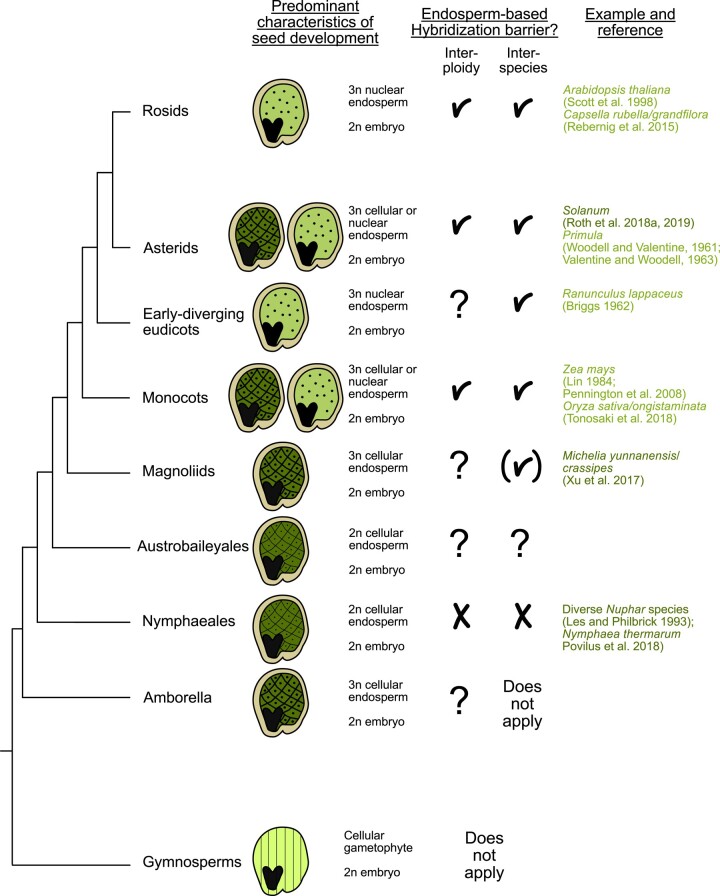
Widespread occurrence of EBBs. The presence of endosperm corresponds closely with the emergence of robust hybridization barriers. In gymnosperms, embryo development is supported by the enlarged female gametophyte (denoted by parallel lines), whereas angiosperms have successfully integrated fertilization with the formation of a nourishing endosperm tissue. The endosperm may initiate as a cellular structure, as seen in ANA (Amborella, Nymphaeales, and Austrobaileyales) species that are sister lineages to all other angiosperms (indicated by the checkerboard pattern), or as a coenocyte where initial nuclear divisions are not synchronized with cellularization (depicted by the dotted pattern). References for each category of barriers are provided; for a more comprehensive exploration of reported barriers, we direct readers to Coughlan (2023). References in light green refer to barriers occurring in nuclear-type endosperm. Dark green references refer to cellular-type barriers. Checkmarks symbolize presence of EBBs. Brackets symbolize that insufficient data are available to determine whether the endosperm is affected in hybrid seeds. Question marks symbolize that no data are available yet.

Numerous studies conducted in the last century have reported the failure of producing viable crop hybrids when crossing related plant species due to the widespread occurrence of seed lethality (Brink and Cooper 1947; Williams and White 1976; Gill and Waines 1978; Johnston and Hanneman Jr 1982; Sukno et al. 1999). By histological observations and in vitro embryo rescue experiments, these studies identified abnormal endosperm development as the primary cause of hybrid seed lethality in response to interspecies crosses (Brink and Cooper 1947; Valentine and Woodell 1963; Sukno et al. 1999; Dinu et al. 2005; Roy et al. 2011). This phenomenon’s prevalence across diverse plant taxa (Fig. 1; Coughlan 2023) suggests that endosperm-based hybrid seed lethality (later on referred to as endosperm-based barriers [EBBs]) represents a substantial mode of reproductive isolation in flowering plants.

Similar to interspecies crosses, crosses involving plants with differing numbers of chromosome sets (ploidy) also result in seed lethality, with the outcome dependent on the direction of the cross. To describe this phenomenon, we will employ the term “paternal excess seeds” for seeds resulting from crosses where the paternal parent has a higher ploidy, while “maternal excess seeds” refer to those resulting from the reciprocal cross direction. Both types of seeds, whether they exhibit maternal or paternal excess, show abnormalities in endosperm development. In species characterized by the nuclear mode of endosperm development, paternal excess leads to a delay in endosperm cellularization, whereas maternal excess has the opposite effect, causing precocious cellularization (Scott et al. 1998; Pennington et al. 2008; Sekine et al. 2013). In species with the cellular mode of endosperm development, paternal excess seeds are characterized by larger and more vacuolated endosperm cells, while maternal excess seeds have smaller and less vacuolated cells (Woodell and Valentine 1961; von Wangenheim and Peterson 2004). Maternal and paternal excess-like phenotypes have also been observed in interspecies crosses where the ploidy of the parental plants is alike (Brink and Cooper 1947; Sukno et al. 1999; Dinu et al. 2005; Roy et al. 2011; Baek et al. 2016; Oneal et al. 2016; Roth et al. 2018a; Coughlan et al. 2020). These deviations in endosperm development observed in both interploidy and interspecies crosses suggest a common mechanistic basis for these phenomena. This notion gains substantial support from the observation that interspecies EBBs can be overcome by increasing the ploidy of 1 parent, highlighting the involvement of a dosage-sensitive component in both types of EBBs (Johnston and Hanneman Jr 1982; Lafon-Placette et al. 2017; Tonosaki et al. 2018).

The concept of the endosperm balance number (EBN) provides a theoretical framework for understanding the dosage sensitivity of the endosperm and offers a means to assess hybridization success between different species (Johnston et al. 1980). According to this concept, each species possesses an effective ploidy, which may differ from its actual (karyotypic) ploidy. The effective ploidy is determined through test crosses with a defined species, allowing the assessment of cross-compatibility with other species (Johnston et al. 1980; Städler et al. 2021). Only species with similar EBNs can produce viable seeds by maintaining an effective 2:1 maternal-to-paternal ratio in the endosperm, a characteristic feature in most angiosperms (Lin 1984; Scott et al. 1998; Leblanc et al. 2002). From now on, we will refer to maternal or paternal excess crosses when the maternal or paternal parent has the higher effective ploidy, respectively. The focus however will be on paternal excess crosses, as most molecular research has concentrated on unraveling the molecular mechanisms underlying EBBs originating from these specific types of crosses.

## Genomic imprinting: An epigenetic phenomenon underpinning reproductive isolation

The nonreciprocal effects observed in interploidy and interspecies hybridizations defy Mendelian expectations, revealing that parental genomes exert varying influences on offspring. Genomic imprinting, an epigenetic phenomenon that induces alterations in gene expression based on parental origin, explains these nonreciprocal hybridization outcomes. In the context of flowering plants, genomic imprinting primarily occurs within the endosperm and substantially influences its growth (Rodrigues and Zilberman 2015; Satyaki and Gehring 2017; Batista and Köhler 2020).

Imprinted genes are epigenetically modified, a process that typically initiates within the gametes and persists following fertilization. This results in genes that are specifically expressed either maternally (MEGs) or paternally (PEGs). Imprinted genes are enriched for transposable elements (TEs) in their flanking regions (Gehring et al. 2009; Wolff et al. 2011; Rodrigues et al. 2013; Pignatta et al. 2014; Hatorangan et al. 2016), with many of them subject to regulation by the DNA glycosylase DEMETER (DME). DME excises 5′-methylcytosine, and its activity in the central cell of the female gametophyte leads to the activation of maternal alleles for certain MEGs that remain hypomethylated after fertilization (Choi et al. 2002; Pignatta et al. 2014; Park et al. 2016). Conversely, for PEGs, DNA hypomethylation facilitates Polycomb Repressive Complex2 (PRC2)–directed trimethylation at lysine 27 of histone H3 (H3K27me3), resulting in the suppression of maternal alleles of PEGs (Moreno-Romero et al. 2016, 2019).

The relationship between imprinted genes and flanking TEs initially led to the hypothesis that imprinting primarily functions to repress TEs (Gehring et al. 2009; Hsieh et al. 2009). However, recent research challenges this notion, revealing that numerous TEs harbor transcription factor binding sites. This discovery implies that DME-mediated demethylation plays a vital role in kick-starting gene expression by granting access to transcription factors (Schmitz et al. 2013; Batista et al. 2019b; Khouider et al. 2021). Hence, it appears that endosperm imprinting results from a process enabling the transcriptional activation of MEGs through DME-mediated demethylation, with some of these MEGs being crucial for seed development. Notably, *MEDEA* and *FERTILIZATION INDEPENDENT SEED2* (*FIS2*), encoding subunits of the PRC2, are essential MEGs that subsequently contribute to the suppression of the maternal alleles of PEGs (Zhang et al. 2014; Moreno-Romero et al. 2019).

Aside from the molecular explanations of how genomic imprinting is established, there are many considerations on the possible evolutionary advantages of this phenomenon. Among those, the “parental conflict” or “kinship theory” (Trivers 1974; Haig and Westoby 1989, 1991) is the most widely accepted evolutionary theory that provides a theoretical framework for the selection of imprinted genes. According to this theory, conflict arises because siblings compete for limited maternal resources. Since the maternal parent is equally related to its offspring, balanced provisioning of the progeny maximizes maternal fitness (Haig and Westoby 1989). Multiple paternity increases this conflict, since each offspring is more closely related to itself than to its siblings (Trivers 1974; Haig and Westoby 1989, 1991). This conflict drives the evolution of paternally derived alleles that enhance resource acquisition, while maternally derived alleles are selected to equalize nutrient allocation (Queller 1983; Haig and Westoby 1989, 1991). Consequently, MEGs and PEGs are predicted to be frequently associated with opposing functions in the endosperm, either restricting or promoting endosperm growth (Haig and Westoby 1989, 1991; Costa et al. 2012). Supporting this theory, an increased maternal genome dosage leads to reduced endosperm growth and smaller seeds, whereas an increased paternal genome dosage produces the opposite effect (Brink and Cooper 1947; Scott et al. 1998; von Wangenheim and Peterson 2004; Stoute et al. 2012; Sekine et al. 2013; Rebernig et al. 2015; Roth et al. 2019). Interestingly, the chalazal region of the endosperm is particularly affected by changes in parental genome dosage (Scott et al. 1998; Sandstedt and Sweigart 2022). This region of the endosperm serves as an interface between maternal and endosperm tissues, playing a pivotal role in determining the extent of nutrient flow from the maternal plant to the embryo and endosperm (Povilus and Gehring 2022). PEGs are most highly expressed in the chalazal endosperm, pointing at a functional role of PEGs in regulating nutrient transfer by affecting the growth of the chalazal endosperm (Picard et al. 2021).

The cross-direction–dependent seed phenotypes observed in interspecies and interploidy hybridizations have led to the hypothesis that imprinted genes play a causal role in establishing EBBs (Haig and Westoby 1989, 1991; Moore 2001; Gutierrez-Marcos et al. 2003). Genetic studies provide support for this notion, demonstrating that mutations in several PEGs can suppress triploid seed abortion (referred to as triploid block) in Arabidopsis (Kradolfer et al. 2013b; Wolff et al. 2015; Huang et al. 2017; Batista et al. 2019b). Suppression of the triploid block by mutations in PEGs consistently corresponds with the restoration of endosperm cellularization (Wolff et al. 2015; Batista et al. 2019b), underscoring the critical role of endosperm cellularization in embryo growth. Many PEGs associated with the triploid block are generally highly upregulated in triploid seeds. However, this upregulation is not attributed to a breakdown of imprinting but rather to enhanced expression of the active paternal alleles (Batista et al. 2019b). In contrast, seed arrest in interspecies hybrids is associated with disruption of imprinting in hybrid endosperm (Josefsson et al. 2006; Burkart-Waco et al. 2015; Florez-Rueda et al. 2016; Kinser et al. 2021). Nevertheless, while the cause for the deregulation of imprinted genes in interploidy and interspecies hybrids may differ, the consequences of their deregulation are similar and lead to shared endosperm phenotypes with lethal consequences.

### Small RNAs and DNA methylation in the establishment of reproductive isolation

Paternally inherited mutations in the RNA-directed DNA methylation (RdDM) pathway suppress the triploid block (Erdmann et al. 2017; Martinez et al. 2018; Satyaki and Gehring 2019; Wang et al. 2021), similar to mutants in the maintenance methyltransferase MET1 or chemically induced hypomethylation (Schatlowski et al. 2014; Huc et al. 2021). The RdDM pathway initiates with the activity of RNA polymerase IV (Pol IV), which generates short transcripts that are transformed into double-stranded RNAs by RNA-dependent RNA POLYMEREASE 2. These double-stranded RNAs are then cleaved into small interfering RNAs (siRNAs) of 21 to 24 nucleotides (nts) by DICER-like proteins that can be loaded into different ARGONAUTE proteins (Cuerda-Gil and Slotkin 2016). In pollen, Pol IV is required for generating 21- to 22-nt epigenetically activated siRNAs (easiRNAs) as well as 24-nt siRNAs. easiRNAs originate from TEs within the vegetative nucleus and are likely transported to sperm cells afterwards (Slotkin et al. 2009; Martinez et al. 2016). Notably, the formation of easiRNAs relies on microRNA845a (miR845a) and miR845b, 2 abundant pollen miRNAs that target long terminal repeat retrotransposons at their reverse transcription primer binding site. In line with the importance of easiRNAs in the triploid block, a mutation in *MIR845b* can alleviate this block (Borges et al. 2018).

Pol IV–generated siRNAs recruit the de novo DOMAINS REARRANGED METHYLTRANSFERASE2, which establishes DNA methylation in all sequence contexts (Chow et al. 2020). Interestingly, inbreeding of RdDM mutants enhances their ability to suppress the triploid block and is linked with a gradual decrease in DNA methylation over generations. In contrast to other RdDM pathway mutants, *nrpd1* (affected in the large subunit of Pol IV, NUCLEAR RNA POLYMERASE D1) exhibits a suppressive effect in the first generation of inbreeding (Wang et al. 2021), suggesting that the loss of easiRNAs and paternal DNA methylation can independently suppress the triploid block. Recent findings further support this notion, revealing that the depletion of easiRNAs in the vegetative cell of pollen (but not in sperm cells) can suppress the triploid block (Pachamuthu et al. 2023). Whether paternally delivered easiRNAs are causally responsible for DNA methylation changes in the endosperm as previously proposed (Martinez et al. 2018) remains to be investigated.

Despite that paternally inherited *nrpd1* can suppress the triploid block, the transcriptome changes in triploid seeds inheriting *nrpd1* through pollen remain relatively mild. Among the genes that continue to be upregulated in triploid *nrpd1* seeds are several PEGs previously identified to suppress the triploid block when mutated (Martinez et al. 2018; Satyaki and Gehring 2019). There are 2 possible hypotheses to explain this data: either Pol IV operates in a parallel pathway to the known suppressor genes or it acts downstream of these genes. Further research is required to distinguish between these hypotheses.

A link between the dosage of siRNAs and gene deregulation in hybrid endosperm has also been established in *Solanum*, where reciprocal hybrid seeds exhibit reduced siRNA levels corresponding with increased gene expression at specific loci potentially targeted by siRNAs (Florez-Rueda et al. 2021). Similarly, *Capsella* paternal excess hybrid endosperm shows strongly depleted DNA methylation, which associates with chromatin decondensation (Dziasek et al. 2021) and loss of siRNAs that depend on maternal Pol IV function (Dziasek et al. 2023). The loss of chromatin condensation in *Capsella* hybrid endosperm is associated with increased gene expression, particularly near heterochromatic pericentromeric regions, including many targets of type I MADS-box transcription factors (referred to as AGAMOUS-LIKE TFs or AGLs). These AGLs are consistently upregulated in paternal excess interploidy and interspecies hybrids (Josefsson et al. 2006; Erilova et al. 2009; Ishikawa et al. 2010; Burkart-Waco et al. 2013; Sekine et al. 2013; Rebernig et al. 2015; Roth et al. 2019; Bjerkan et al. 2020; Dziasek et al. 2021). AGL upregulation is likely causally relevant in establishing EBBs, since mutants in several *AGLs* show weakened interploidy and interspecies barriers (Josefsson et al. 2006; Walia et al. 2009; Hehenberger et al. 2012; Batista et al. 2019b). Maternal siRNAs inversely correspond with *AGL* expression (Lu et al. 2012; Kirkbride et al. 2019; Dziasek et al. 2023), suggesting a connection between the loss of maternal siRNAs, DNA methylation, and increased expression of *AGLs*. These Pol IV–dependent siRNAs exhibit a proportional increase corresponding to the ploidy of the maternal genome. Consequently, maternal excess in Arabidopsis leads to heightened accumulation of siRNAs, while paternal excess results in diminished siRNA abundance, corresponding with increased *AGL* expression (Lu et al. 2012; Kirkbride et al. 2019). Notably, maternal Pol IV–dependent siRNAs, originating from a few loci in high abundance, have been observed across various flowering plant species. The loci responsible for producing these siRNAs appear to evolve rapidly (Grover et al. 2020), hinting at a potential role in establishing reproductive barriers between species. Where exactly maternal Pol IV–dependent siRNAs are produced remains to be established; while they have been proposed to be predominantly maternally produced in *Brassica* (Grover et al. 2018, 2020), biparental production was found in Arabidopsis (Satyaki and Gehring 2022). Collectively, alterations in the dosage of maternal and paternal Pol IV–dependent siRNAs impact EBBs. However, dependent on their parental origin, these siRNAs are generated from distinct loci and influence different targets, in line with their antagonistic effects on gene expression and endosperm development (Satyaki and Gehring 2022).

## Epigenetic regulators as key players in overcoming the triploid block

**Figure 2.**
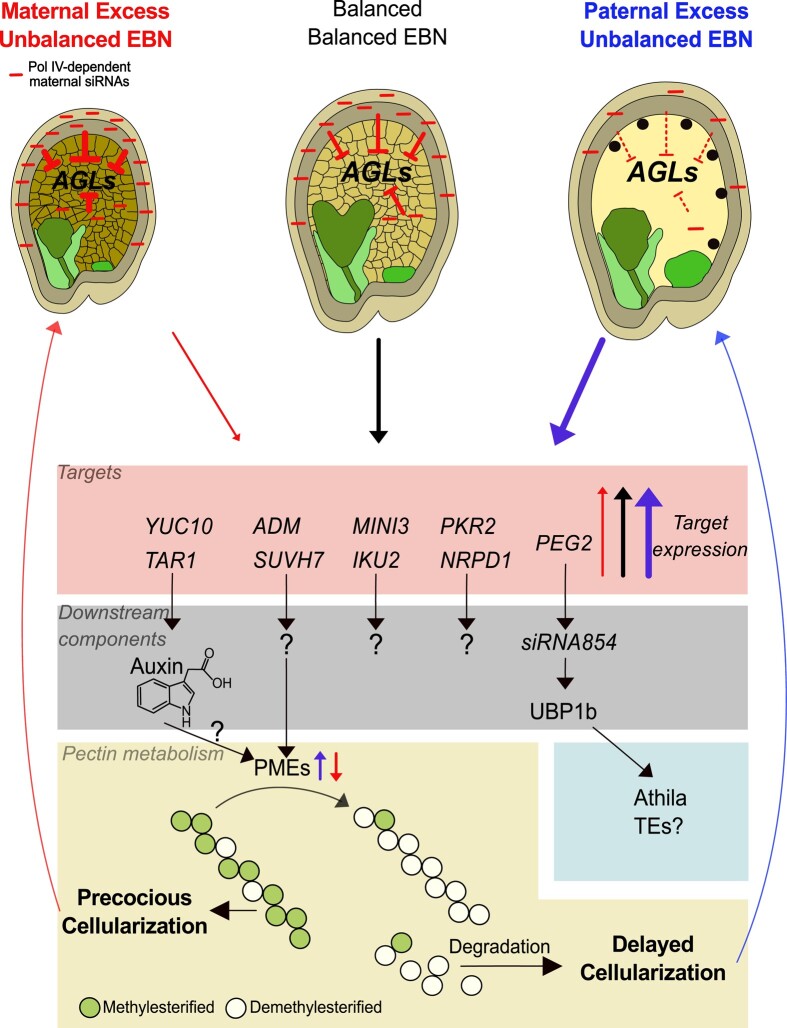
Molecular pathways underlying endosperm-based interploidy and interspecies barriers. Maternal Pol IV–dependent siRNAs act as dosage-dependent repressors of AGL MADS-box genes. Increased dosage of siRNAs in maternal excess crosses corresponds with strong AGL repression (reflected by blunt-ended arrows; thickness of the arrows corresponds to intensity of repression) and early endosperm cellularization, while decreased siRNA dosage in paternal excess crosses corresponds with increased AGL abundance and late endosperm cellularization. High and low AGL dosage causes increased or decreased expression of downstream AGL targets, respectively, including auxin biosynthesis genes. Downstream direct or indirect targets of this signaling cascade are genes involved in pectin metabolism, including pectin methylesterase (PME) genes. Demethylesterification exposes pectin to pectin–degrading enzymes, thereby establishing a connection between the expression level of *AGLs* and the timing of endosperm cellularization. Abundance of AGLs and their downstream targets in response to hybridization are depicted by red (low), gray (balanced), and blue (high) arrows. Light and dark green endosperm regions mark micropylar and chalazal domains, respectively. ADM, ADMETOS; AGLs, AGAMOUS-LIKE genes; EBN, endosperm balance number; IKU2, HAIKU2; MINI3, MINISEED3; NRPD1, NUCLEAR RNA POLYMERASE D1; PEG2, PATERNALLY EXPRESSED GENE2; UBP1, OLIGOURIDYLATE BINDING PROTEIN 1B; PKR2, PICKLE RELATED2; SUVH7, SU(VAR)3–9 HOMOLOG 7; TAR1, TAA1-RELATED1; TEs, transposable elements; YUC10, YUCCA10.

PHERES1 (PHE1) is a highly upregulated AGL in triploid Arabidopsis seeds that directly binds to the promoter region of PEGs, many of those also being highly upregulated in triploid seeds (Batista et al. 2019b). Among them, *ADMETOS* (*ADM*), *SU(VAR)3–9 HOMOLOG 7* (*SUVH7*), *PICKLE RELATED2* (*PKR2*), and *PEG2* have been identified to suppress the triploid block when mutated (Kradolfer et al. 2013a; Wolff et al. 2015; Huang et al. 2017; Fig. 2). ADM physically interacts with SUVH9 and various AT HOOK-LIKE (AHL) proteins. Mutations in *suvh9* and *ahl10*, similar to *adm*, effectively suppress the triploid block, suggesting the 3 genes act in the same pathway (Jiang et al. 2017). SUVH9 has the capacity to bind to methylated DNA, facilitating the recruitment of DNA-dependent RNA polymerase V (Pol V) to chromatin (Johnson et al. 2014; Liu et al. 2014b). Simultaneously, AHLs bind to AT-rich chromosomal regions, inducing increased levels of H3K9me2 via unknown mechanisms (Yun et al. 2012; Xu et al. 2013). Notably, in triploid seeds, ADM-dependent mislocalization of H3K9me2 is targeted at AT-rich small TEs, inversely correlating with CHH methylation. Recent work has shown that the catalytic domain of SUVH9 is essential for its function (Parent et al. 2021), suggesting that AHL10-ADM-SUVH9 may collectively recruit H3K9me2 to TEs in the endosperm, potentially leading to the ectopic recruitment of H3K9me2 in triploid seeds (Jiang et al. 2017). This ectopic recruitment of H3K9me2 inversely correlates with CHH methylation, suggesting that AHL10-ADM-SUVH9–mediated H3K9me2 at specific TEs opposes the RdDM pathway in triploid seeds, in line with previous reports on the inhibitory effect of H3K9me2 on the RdDM pathway (Schoft et al. 2009; Zemach et al. 2013; Gent et al. 2014).

The roles of SUVH7 and PKR2 in relation to the triploid block are yet to be fully elucidated. Given that PKR2 shares homology with PICKLE (PKL), which acts in the RdDM pathway by supporting the accumulation of Pol V–dependent transcripts (Yang et al. 2017), it is plausible that PKR2 serves a related function. Similar to other mutants in the RdDM pathway (Wang et al. 2021), the suppressive effect of *pkr2* appears to require several generations of inbreeding (Huang et al. 2017). The *pkr2* mutant was not initially identified as a suppressor when tested after introgression into the meiotic mutant *omission of second division 1* (*osd1*; Wolff et al. 2015), in contrast to its suppressive effect following inbreeding (Huang et al. 2017).

The suppressive effect of *peg2* on the triploid block has been linked to the ability of the *PEG2* transcript to sequester the TE-derived *siRNA854* (Wang et al. 2018). This suggests that a reduced abundance of *siRNA854* has functional consequences for endosperm development. *SiRNA854* regulates the abundance of the stress granule–associated protein OLIGOURIDYLATE BINDING PROTEIN 1B (UBP1b) by translational repression (McCue et al. 2012), which is consistent with *ubp1b* partially suppressing the triploid block (Wang et al. 2018). UBP1b has been proposed to play a role in the translational repression of *Athila* retrotransposon transcripts (McCue et al. 2012). However, whether this mechanism is mechanistically connected to the triploid block requires further investigation.

Collectively, many of the triploid block suppressors identified in Arabidopsis encode for chromatin regulators with potential roles in TE silencing or the establishment of heterochromatin (Wolff et al. 2015; Jiang et al. 2017; Yang et al. 2017; Satyaki and Gehring 2019; Wang et al. 2021). Similarly, many genes responsible for hybrid incompatibility in *Drosophila* encode for dosage-sensitive heterochromatin-interacting proteins or components of the PIWI pathway that silences TEs (Brideau et al. 2006; Bayes and Malik 2009; Thomae et al. 2013; Parhad et al. 2017). In *Drosophila*, TEs have been implicated in causing intraspecies incompatibility (Castillo and Moyle 2022). Nevertheless, like in plants, the mechanisms through which TEs and deregulated chromatin regulators induce lethality remain to be fully established.

## The role of auxin in antagonizing endosperm cellularization

**Figure 3.**
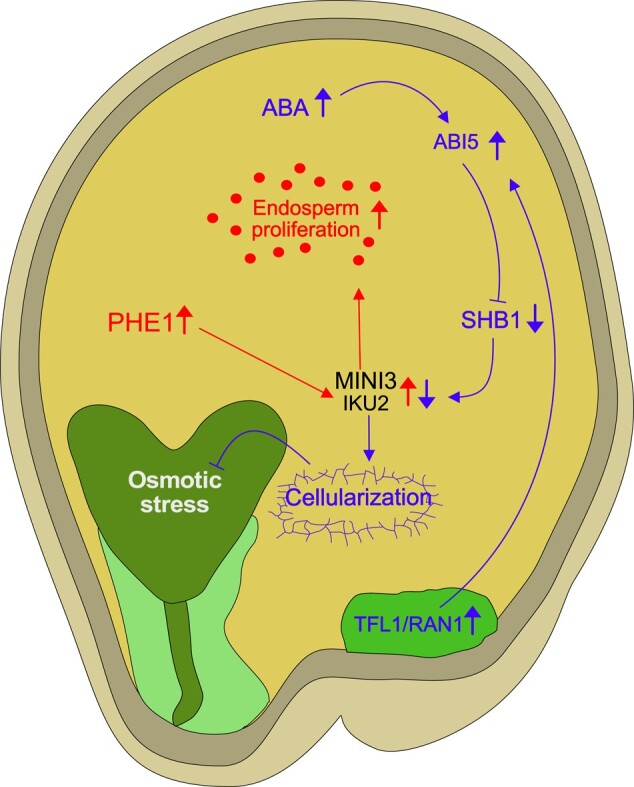
Regulation of endosperm cellularization by antagonizing activities of auxin and ABA. ABA activates the downstream transcription factor ABI5, which, in turn, is stabilized by mobile factors such as TFL1, which is transported from the chalazal region to the peripheral endosperm. The mobilization of TFL1 is facilitated by RAN1. ABI5, under the influence of ABA, exerts its regulatory function by downregulating *SHB1*. SHB1 is a positive regulator of 2 critical genes, *MINI3* and *IKU2*, both of which are direct targets of PHE1. MINI3 and IKU2 act as positive regulators of endosperm proliferation (symbolized by red dots). The repression of *MINI3* and *IKU2* via the ABA pathway likely promotes endosperm cellularization. Failure of endosperm cellularization can trigger an osmotic stress response in the embryo. Red arrows in this network represent the auxin-PHE1 pathway, and blue arrows highlight the ABA signaling pathway. Upward-pointing arrows reflect increased expression or activity, while downward-pointing arrows reflect decreased expression or activity. Arrows with blunt ends reflect repressive activity. Light and dark green endosperm regions mark micropylar and chalazal domains, respectively. ABI5, ABSCISIC ACID-INSENSITIVE5; IKU2, HAIKU2; MINI3, MINISEED3; PHE1, PHERES1; RAN1, Ras-related nuclear GTPase1; SHB1, SHORT HYPOCOTYL UNDER BLUE1; TFL1, TERMINAL FLOWER1.

Beyond its interactions with various epigenetic regulators, PHE1 also binds to auxin biosynthesis genes *YUCCA10* (*YUC10*) and *TRYPTOPHAN AMINOTRANSFERASE RELATED 1* (*TAR1*). Activated by PHE1, *YUC10* and *TAR1* are highly overexpressed in triploid seeds, concomitant with increased auxin activity (Batista et al. 2019a, 2019b; Fig. 3).

Elevated auxin levels wield a negative impact on endosperm cellularization, establishing a direct link between the upregulation of *PHE1*, increased auxin production, and the failure of endosperm cellularization (Batista et al. 2019a). Interestingly, increasing auxin levels, specifically in the seed coat, also block endosperm cellularization, implicating the seed coat as a target of auxin action that indirectly impacts endosperm cellularization. Notably, in triploid seeds, increased auxin activity is primarily detected in the seed coat rather than the endosperm, hinting at auxin’s production in the endosperm and its subsequent transportation to the seed coat, drives seed coat expansion (Batista et al. 2019a). AGL62 emerges as a crucial player in regulating auxin transport from the endosperm to the seed coat, potentially by modulating the expression of the ABCB-type transporter gene *PGP10* (Figueiredo et al. 2016) and regulating auxin biosynthesis (Guo et al. 2022). Supporting this notion is the observation that the *agl62* mutant exhibits partial suppression of EBBs (Walia et al. 2009; Hehenberger et al. 2012). This intricate regulation of auxin transport between the endosperm and seed coat potentially underpins the mechanism behind the triploid block.

The suppressive effect of *transparent testa* (*tt*) mutants, affecting flavonoid biosynthesis in the innermost layer of the inner integument, offers another potential link between auxin and the triploid block. Flavonoids impact polar auxin transport by interacting with auxin transporters such as PGPs (Peer and Murphy 2007). Several mutants within the TT pathway, including *transparent testa glabra2* (*ttg2*), *tt4*, *tt7*, *tt8*, and *tt13*, exhibit the ability to suppress the triploid block (Dilkes et al. 2008; Scott et al. 2013; Doughty et al. 2014; Zumajo-Cardona et al. 2023), although the precise mechanisms remain elusive. Intriguingly, the efficiency of this suppression varies among the *tt* mutants, with *tt8* causing complete suppression, while others have a milder effect (Zumajo-Cardona et al. 2023), hinting at the involvement of multiple pathways regulated by *TT* genes.

A plausible model suggests that type I MADS-box transcription factors, such as PHE1 and AGL62, govern auxin homeostasis within the endosperm. This regulation includes binding to the promoter regions of auxin biosynthesis genes, resulting in increased auxin production in direct proportion to the increased dosage of AGLs (Figueiredo et al. 2016; Batista et al. 2019a). Auxin reduces tissue rigidity in the shoot apical meristem by initiating the demethylesterification of homogalacturonan, a component of pectin. This process renders it susceptible to enzymatic depolymerization, facilitating gel formation and degradation (Braybrook and Peaucelle 2013; Wormit and Usadel 2018). Genes linked to demethylesterification and degradation of pectin are significantly overexpressed in triploid hybrid seeds that fail to undergo endosperm cellularization, suggesting that auxin regulates pectin metabolism with consequences on endosperm cellularization (Rebernig et al. 2015; Wolff et al. 2015).

## Abscisic acid facilitates endosperm cellularization and enhances triploid seed viability

The precise timing of endosperm cellularization represents a critical factor for embryo survival, a phenomenon consistently disrupted in interploidy and interspecies hybrids (Muntzing 1933; Brink and Cooper 1947; Woodell and Valentine 1961; Scott et al. 1998; Dilkes et al. 2008; Hehenberger et al. 2012). The reasons underpinning the necessity of endosperm cellularization for embryo survival have long been a matter of conjecture. One proposed hypothesis suggests that the failure of endosperm cellularization results in an inadequate supply of sucrose to the embryo, as the central vacuole remains the primary resource sink in the seed (Lafon-Placette and Kohler 2014). However, recent research suggests an alternative perspective: embryos surrounded by noncellularized endosperm elicit an embryo-specific osmotic stress response akin to the seed maturation process. This implies that endosperm cellularization serves to protect the embryo from desiccation, a process typically occurring during maturation (Kozaki and Aoyanagi 2022; Xu et al. 2023). Supporting this view, mutations in abscisic acid (ABA) biosynthesis and signaling exacerbate the triploid block, while elevated endogenous ABA levels induce endosperm cellularization and suppress the arrest of embryo growth (Xu et al. 2023). Nevertheless, whether the suppressive effect of increased ABA is a consequence of ABA-induced endosperm cellularization or an enhanced tolerance of the embryo to desiccation remains to be established.

Interestingly, exposure to cooler temperatures during early seed maturation leads to ABA retention in the endosperm upon desiccation (Chen et al. 2021). Similarly, cold treatment enhances the viability of Arabidopsis hybrid seeds impaired in endosperm cellularization (Bjerkan et al. 2020), suggesting that the survival of hybrid seeds in response to cold is linked to elevated ABA levels.

The process of ABA-mediated endosperm cellularization hinges on the downregulation of *SHORT HYPOCOTYL UNDER BLUE1* (*SHB1*) by ABSCISIC ACID-INSENSITIVE5 (ABI5). ABI5-deficient plants exhibit delayed endosperm cellularization and develop enlarged seeds (Cheng et al. 2014), which, notably, intensify the triploid block (Xu et al. 2023). SHB1, in turn, upregulates *MINISEED3* (*MINI3*) and the LRR receptor kinase gene *HAIKU2* (*IKU2*), both of which are direct targets of PHE1 and exert a positive influence on endosperm proliferation (Garcia et al. 2003; Luo et al. 2005; Batista et al. 2019a).

Recent investigations have unveiled the interaction between ABI5 and TERMINAL FLOWER1 (TFL1), a mobile regulator generated in the chalazal endosperm that then relocates to the peripheral endosperm to stabilize ABI5 (Zhang et al. 2020). The movement of TFL1 is modulated by Ras-related nuclear GTPases (RANs) previously implicated in seed size regulation (Liu et al. 2014a). A plausible model posits that ABA activates ABI5, which is subsequently stabilized by TFL1 and RAN1. ABI5 then represses *SHB1*, which in turn restrains *IKU2* and *MINI3*, recognized regulators of endosperm proliferation (Fig. 3). This intricate network is juxtaposed with PHE1, which binds to *IKU2* and *MINI3*, likely promoting their transcription (Batista et al. 2019b).

These findings underscore the convergence of multiple pathways on MINI3 and IKU2, with 1 pathway positively regulated by auxin and PHE1 and the other negatively regulated by ABA, ABI5, and SHB1. While the loss of MINI3 function does not impact the triploid block (Batista et al. 2019a), a mutation in the MINI3 interaction partner IKU1 enhances the survival of Arabidopsis interspecies hybrids (Burkart-Waco et al. 2013), suggesting that the dosage of components within the IKU1-IKU2-MINI3 pathway plays a pivotal role in erecting hybridization barriers by affecting endosperm cellularization.

### Understanding the relevance and drivers of endosperm-mediated reproductive barriers

As elaborated in the preceding sections, the establishment of reproductive barriers mediated by the endosperm is intricately linked to the dysregulation of imprinted genes, particularly PEGs (Wolff et al. 2015; Florez-Rueda et al. 2016; Batista et al. 2019b; Kinser et al. 2021). In line with theoretical expectations, species characterized by outbreeding tendencies are presumed to experience heightened parental conflict, thus predicting an increased abundance or elevated expression of imprinted genes (Brandvain and Haig 2005). Indeed, empirical evidence supports this prediction, as outbreeding species tend to exhibit higher numbers or levels of PEGs compared to their inbreeding relatives (Klosinska et al. 2016; Lafon-Placette et al. 2018).

This augmented prevalence and expression of PEGs in outbreeders could account for the robust EBBs that often manifest between inbreeding and outbreeding species. This phenomenon has been theoretically framed as the “weak inbreeder/strong outbreeder” (WISO) hypothesis (Brandvain and Haig 2005). The validity of the WISO hypothesis is substantiated by extensive evidence, with documented EBBs observed between inbreeders and outbreeders in plant genera such as Arabidopsis, *Arabis*, and *Capsella* (Brandvain and Haig 2005; Lafon-Placette and Kohler 2016; Lafon-Placette et al. 2018; İltaş et al. 2021; Petrén et al. 2023). Similar to differences in breeding mode, also differences in effective population size can lead to strong reproductive barriers. A decrease in population size is anticipated to diminish conflicts among siblings as mating partners become highly related, akin to the impact of selfing. Correspondingly, within outbreeding species of *Solanum* and *Mimulus*, a smaller population size (reflected by lower nt diversity) is associated with a lower EBN (Roth et al. 2018a, 2019; Coughlan et al. 2020; Sandstedt et al. 2021; Sandstedt and Sweigart 2022). Interestingly, in *Solanum*, out of 3 tested species, the one with the highest EBN has the highest number of PEGs (Roth et al. 2018b, 2019). This finding underscores a potential connection between number of PEGs, intensity of parental conflict, and strength of EBBs.

Genomic imprinting is a dynamically evolving phenomenon, characterized by low conservation of imprinted loci across species (Waters et al. 2013; Pignatta et al. 2014; Hatorangan et al. 2016; Klosinska et al. 2016; Chen et al. 2018). In alignment with the hypothesized causal role of PEGs in the establishment of reproductive barriers, EBBs themselves exhibit a propensity for rapid evolution (Rebernig et al. 2015; Oneal et al. 2016; Roth et al. 2018a; Coughlan et al. 2020; İltaş et al. 2021). The exploration of the functional role of imprinted genes in the context of interspecies hybridization barriers promises to be an exciting avenue for future research.

Another compelling research direction lies in the relationship between the strength of EBBs and the mode of endosperm development. Notably, robust barriers are encountered in species characterized by both nuclear and cellular endosperm development, suggesting that the mode of endosperm development itself does not substantially influence barrier strength. Furthermore, the ploidy level of the endosperm does not appear to be the decisive factor, as evidenced by the robust interploidy barriers observed in species like evening primrose (*Oenothera hookeri*), which have a diploid endosperm (von Wangenheim 1962). Instead, the key determinant seems to be the role of the endosperm in provisioning the embryo, a role that varies among species. In species where the endosperm plays a pivotal role in embryo provisioning, strong EBBs tend to be prevalent. Conversely, barriers are generally weaker in species where the endosperm has a relatively minor role in this context, such as in Nymphaeales, which rely on the maternal sporophytic nucellus for seed storage (Les and Philbrick 1993; Povilus et al. 2018). An intriguing question for future investigation pertains to whether the presence of weak EBBs is a recurring pattern among angiosperms with reduced endosperms.

In conclusion, our comprehension of the genetic underpinnings of EBBs has made substantial progress in recent years, shedding light on intriguing facets while simultaneously unveiling additional inquiries (see Outstanding Questions). Tackling these emerging questions collectively as a scientific community promises to be an exciting endeavor for the future.

**Outstanding Questions**

- What is the functional role of paternal Pol IV–dependent siRNAs (easiRNAs) in the establishment of reproductive barriers?
- Are maternal Pol IV–dependent siRNAs transmitted from maternal sporophytic tissues to the endosperm, and what are the underlying mechanisms of this process?
- What are the epistatic relationships among the identified suppressor genes associated with hybridization barriers?
- At what pace do hybridization barriers evolve?

## Dive Curated Terms

The following phenotypic, genotypic, and functional terms are of significance to the work described in this paper:

SUVH9 Gramene: AT4G13460
SUVH9 Araport: AT4G13460

agl62 Gramene: AT5G60440
agl62 Araport: AT5G60440

AHL Gramene: AT5G54390
AHL Araport: AT5G54390

MET1 Gramene: AT5G49160
MET1 Araport: AT5G49160

TT8 Gramene: AT4G09820
TT8 Araport: AT4G09820

MIR845b Gramene: AT4G23375
MIR845b Araport: AT4G23375

PGP10 Gramene: AT1G10680
PGP10 Araport: AT1G10680

TT4 Gramene: AT5G13930
TT4 Araport: AT5G13930

TT7 Gramene: AT5G07990
TT7 Araport: AT5G07990

TTG2 Gramene: AT2G37260
TTG2 Araport: AT2G37260

endosperm AmiGo: PO:0009089

OSD1 Gramene: AT3G57860
OSD1 Araport: AT3G57860

NRPD1 Gramene: AT1G63020
NRPD1 Araport: AT1G63020
